# Comparing Cancer Risk Management between Females with Truncating *CHEK2* 1100delC versus Missense *CHEK2* I157T Variants

**DOI:** 10.3390/genes15070881

**Published:** 2024-07-05

**Authors:** Diego Garmendia, Anne Weidner, Lindsay Venton, Tuya Pal

**Affiliations:** 1 Vanderbilt University Medical Center, 1500 21st Ave. So., Suite 2810, Nashville, TN 37212, USA; diego.l.garmendia.1@vumc.org (D.G.); anne.e.weidner@vumc.org (A.W.); lindsay.a.venton@vumc.org (L.V.); 2 Vanderbilt-Ingram Cancer Center, 2220 Pierce Ave, Nashville, TN 37232, USA

**Keywords:** *CHEK2*, germline pathogenic/likely pathogenic variant, 1100delC, I157T, breast cancer, cancer risk, cancer risk management, over-screening, over-treatment

## Abstract

Breast cancer (BC) risks imparted by *CHEK2* c.1100delC (“1100delC”) germline pathogenic/likely pathogenic variant (GPV) are 20–30%, compared to *CHEK2* c.470T>C (“I157T”) GPV with <20%, leading to different breast screening recommendations through MRI. We compared cancer risk management (CRM) across these two GPVs. Study participants were adult females with an 1100delC or I157T GPV drawn from the Inherited Cancer Registry (ICARE) across the United States. Cancer history, clinical characteristics, and CRM were compared using chi-squared tests, t-tests, and logistic regression. Of 150 *CHEK2* carriers, 40.7% had BC, with a mean age of 50. Comparing 1100delC and I157T GPVs, there were no differences in rates of (1) breast MRI among those with (65.2% versus 55.6% of 23 and 9; *p* = 0.612) and without (44.0% versus 44.8% of 50 and 29; *p* = 0.943) BC; (2) risk-reducing mastectomy among those with (50% versus 38.9% of 46 and 15; *p* = 0.501) and without (13.8% versus 6.5% of 58 and 31; *p* = 0.296) BC; and (3) risk-reducing salpingo-oophorectomy among those with (24.2% versus 22.2% of 45 and 18; *p* = 0.852) and without (17.5% versus 16.7% of 57 and 30; *p* = 0.918) BC. The results suggest over-screening with breast MRI among *CHEK2* I157T GPV carriers and possible overuse of risk-reducing surgeries among *CHEK2* carriers.

## 1. Introduction

Germline pathogenic/likely pathogenic variants (GPVs) in *CHEK2* are typically associated with a moderately increased risk of breast cancer in females [1,2,3,4,5,6,7], generally in the range of 20–30% lifetime risk of breast cancer and a relative risk (RR) of >2 [4,7,8,9]. Given that cancer risk management guidelines for breast cancer are anchored to the level of risk, a lifetime breast cancer risk of 20% or greater among females meets the threshold for an annual breast MRI in the United States [10]. Thus, the identification of *CHEK2* is important, given its impact on cancer risk management [11]. Specifically for *CHEK2*, MRI screening is initiated annually starting between ages 30–35, with annual mammograms added starting at age 40 per the current National Comprehensive Cancer Network (NCCN) guidelines [12]. Risk-reducing mastectomy and risk-reducing salpingo-oophorectomy are generally not recommended for *CHEK2* GPVs [10].

The original study to demonstrate that *CHEK2* was associated with increased female breast cancer risk was based on the truncating European founder GPV, c.1100del (p.Thr367fs), herein referred to as “1100delC”, showing the risk to be around two-fold [13], with subsequent studies confirming this original estimate [7,14]. In contrast to the typical moderate risks attributed to *CHEK2* GPVs, primarily based on this truncating *CHEK2* 1100delC founder GPV, there is another common European founder missense GPV, c.470T>C (p.Ile157Thr), herein referred to as “I157T” [15], which imparts a breast cancer risk of less than 20% and RR ~ 1.3 [14,16]. Consequently, females with a *CHEK2* I157T GPV do not reach the threshold for high-risk breast cancer screening solely based on the presence of this GPV.

Given the ongoing refinement of genotype/phenotype associations within genes in the context of a clinician workforce with limited proficiency in genetics and rising rates of bilateral mastectomies in the United States, we believe that efforts such as ours are needed to evaluate the nuances of implementation of gene-based care for inherited cancers. This is especially important given the rising rates of genetic testing and the rapidly evolving practice guidelines. The current study sought to compare cancer risk management for breast cancer among females with *CHEK2* 1100delC and *CHEK2* I157T GPVs.

## 2. Materials and Methods

Participants in the present study were drawn from the Inherited Cancer Registry (ICARE) [17], a research registry approved through the Vanderbilt University Institutional Review Board. Through ICARE enrollment, participants are consented and asked to complete a baseline questionnaire, which includes demographic, clinical, and risk factor information, and an authorization for release of medical records to obtain relevant genetics and medical records (i.e., genetic test reports, pedigrees, pathology reports). Participants are also sent a follow-up questionnaire every two years. Consent forms and questionnaires may be completed either online or on paper. Consented participants had genetic testing through a variety of commercial genetic testing laboratories at the discretion of their treating healthcare provider. Criteria for testing were determined by their treating healthcare provider and not dictated by ICARE protocol. ICARE participants are recruited through various means, including (1) referrals from healthcare providers who have partnered with ICARE at various clinical centers across the United States and internationally and (2) directly online through the registry website (http://inheritedcancer.net accessed on 20 March 2024) [17]. Over 6000 participants are currently enrolled in ICARE, of which almost two-thirds have a GPV in an inherited cancer-predisposing gene.

**Figure 1:**
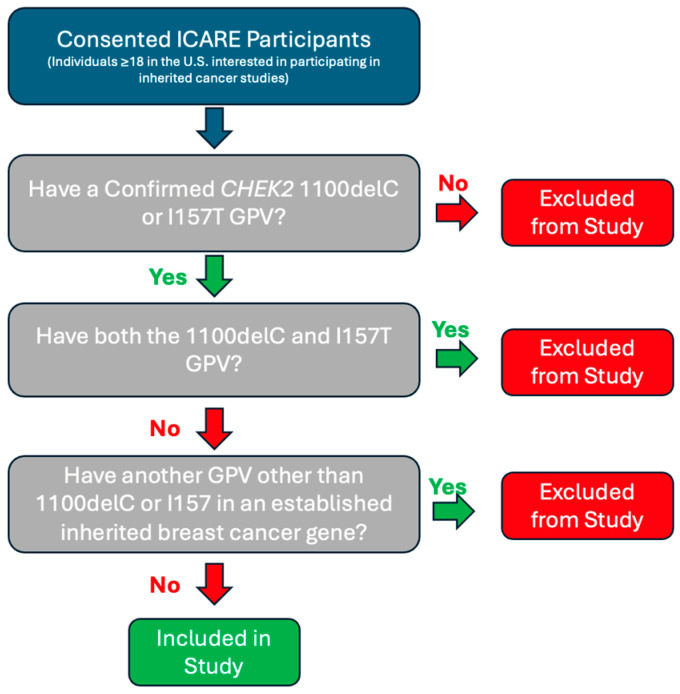
Study Inclusion and Exclusion Criteria.

Individuals enrolled in ICARE with a confirmed *CHEK2* 1100delC or *CHEK2* I157T GPV were included in this study. Excluded individuals included those with GPVs other than *CHEK2* 1100delC or *CHEK2* I157T, individuals with an additional GPV in another established inherited breast cancer gene, and individuals with both *CHEK2* 1100delC and *CHEK2* I157 GPVs. The years of enrollment into ICARE covered July 2011 to May 2023. Figure 1 outlines the inclusion and exclusion criteria for this study.

Demographic and clinical information were summarized and compared between *CHEK2* 1100delC and *CHEK2* I157T carriers using Pearson’s chi-squared tests for categorical variables and t-tests for continuous variables. Pearson’s chi-squared tests were used to compare personal breast cancer history, family history of breast cancer, personal history of other cancer, breast cancer receptor status, breast cancer staging information, and cancer risk management between the two groups. The two *CHEK2* GPV groups (1100delC and I157T) were further categorized into those with and without a personal history of breast cancer, as this variable presents an additional risk factor. The cancer risk management modalities used in this analysis in females with and without a prior breast cancer diagnosis included breast MRIs (in those with remaining breast tissue), prophylactic mastectomy, and prophylactic salpingo-oophorectomy. Breast MRI and risk-reducing surgery were assessed as binary variables (“yes” or “no”) following receipt of genetic test results. Data to assess ongoing compliance with breast MRIs were not available and beyond the scope of the current study. There was no specific time limit to receive the care. The development of breast cancer post-prophylactic mastectomy was also monitored through the use of follow-up questionnaires.

Logistic regression models were used to identify any significant variables (*CHEK2* GPV carrier status, personal history of breast cancer, and family history of breast cancer) that predict the personal history of breast cancer and uptake of breast MRI, risk-reducing mastectomy, and bilateral prophylactic salpingo-oophorectomy.

## 3. Results

Of 150 total females, 104 had the 1100delC variant, and 46 had the I157T variant. Most carriers were non-Hispanic White (95.3%), married or cohabitating (78.0%), college graduates (61.3%), privately insured (73.3%), employed (59.3%), and had an annual income greater than USD 50,000 (66.7%). A summary of the participant demographic information is shown in Table 1.

Overall, 40.7% had a personal history of breast cancer, with a mean age at diagnosis of 50. Personal history of breast cancer was present in 44.2% of 1100delC carriers with a mean age at diagnosis of 49, compared to 32.6% of I157T carriers with a mean age at diagnosis of 55. A summary of additional clinical characteristics across the two groups of GPVs is included in Table 2. No significant differences were found across any clinical variables (all *p* > 0.05).

Rates of uptake of cancer risk management in females with 1100delC compared to I157T were as follows: (1) For breast MRI (among those with at-risk breast tissue), there was 65.2% versus 55.6% (0.612) for those with a personal history of unilateral breast cancer and 44.0% versus 44.8% (*p* = 0.943) without a personal history of breast cancer, respectively; (2) for risk-reducing mastectomy, there was 50.0% versus 38.9% (*p* = 0.501) for those with a personal history of unilateral breast cancer and 13.8% versus 6.5% (*p* = 0.296) for those without a personal history of breast cancer; and (3) for risk-reducing salpingo-oophorectomy, there was 24.4% versus 22.2% (*p* = 0.852) for those with a personal history of unilateral breast cancer and 17.5% versus 16.7% (*p* = 0.918) for those without a personal history of breast cancer. A summary of the cancer risk management practices is also included in Table 2.

The predictability of breast cancer by *CHEK2* GPV carrier status (either 1100delC or I157T) and family history of breast cancer was determined using a logistic regression model. Logistic regression analyses were also utilized to examine different variables that may influence the uptake of breast MRI, bilateral mastectomy, and bilateral prophylactic salpingo-oophorectomy. These variables were *CHEK2* GPV carrier status, personal history of breast cancer, and family history of breast cancer. The logistic regression models showed that only a personal history of breast cancer was a significant predictor of uptake of breast MRI and bilateral mastectomy. There were no significant predictors of breast cancer or the uptake of a bilateral prophylactic salpingo-oophorectomy. Given that *CHEK2* GPV carrier status was not a significant predictor of these cancer risk management practices indicates that uptake of these treatments does not significantly differ between the 1100delC and I157T groups. Table 3 summarizes these results.

*CHEK2* GPV carrier status and family history of breast cancer were also examined as predictors of bilateral prophylactic mastectomy and bilateral prophylactic salpingo-oophorectomy in participants without a personal history of breast cancer. Neither were significant predictors of either risk-reducing surgery. Table 4 describes these data.

Of the 29 participants with a contralateral prophylactic mastectomy (23 1100delC and 6 I157T), none developed breast cancer (mean follow-up time of 5.69 years). Of the 10 participants with a bilateral prophylactic mastectomy (8 1100delC and 2 I157T), none developed breast cancer (mean follow-up time of 5.75 years).

## 4. Discussion

Findings from our study showed that females with the *CHEK2* I157T GPV have similar cancer risk management practices as those with the *CHEK2* 1100delC GPV despite lower breast cancer risks that do not reach the threshold for high-risk breast cancer screening. Furthermore, the presence of risk-reducing mastectomy and similar rates of risk-reducing salpingo-oophorectomy may represent over-treatment among *CHEK2* carriers.

Overall, these findings suggest that females with the *CHEK2* 1100delC GPV and the *CHEK2* I157T GPV are provided with similar cancer risk management recommendations, despite the I157T GPV having a breast cancer risk of less than 20%, which does not meet the threshold at which high-risk breast screening is recommended [14,16]. Thus, despite the difference in guideline recommendations, our findings indicate that females with one of these two GPVs have similar rates of high-risk screening through breast MRI among those with at-risk breast tissue. These findings were seen when comparing females with the two GPVs in subgroups with and without breast cancer through both frequencies and our logistic regression model, suggesting that those with the I157T GPV may receive screening beyond that recommended in current guidelines. Moreover, rates of risk-reducing salpingo-oophorectomy were similar for females with an 1100delC and I157T GPV, both with and without a breast cancer diagnosis, suggesting possible over-treatment among *CHEK2* carriers. No differences in rates of risk-reducing mastectomy in both participants with and without a prior history of breast cancer among the 1100delC group and the I157T group were observed in the logistic regression model. Ultimately, the presence of risk-reducing mastectomy is not recommended solely based on *CHEK2* GPV carrier status, suggesting possible over-treatment among *CHEK2* carriers. Furthermore, only a personal history of breast cancer was a significant predictor for the uptake of bilateral mastectomy, while a family history of breast cancer was not. These findings suggest that the presence of a *CHEK2* GPV influenced the receipt of risk-reducing surgery, thus supporting the possibility of over-treatment in these individuals. Findings through the current study are consistent with prior studies from us and others, through which possible overtreatment has been suggested, particularly among females with moderate penetrance genes [18,19,20]. A study of over 20,000 women with breast cancer identified care beyond guidelines through high rates of risk-reducing surgery, specifically bilateral mastectomy, in women with GPVs in breast cancer-associated genes other than *BRCA1/2* (95% CI, 37.7−48.5%) [18]. This result was further supported by two of our own studies that found high rates of risk-reducing mastectomies (52% [19] and 43% [20]) and risk-reducing salpingo-oophorectomies (37%) among females with moderate penetrance genes, including *CHEK2* [18]. Our findings, in conjunction with prior studies, suggest there is an opportunity to better educate healthcare providers to promote risk-informed care.

While *CHEK2* is generally categorized as a “moderate penetrance gene” for breast cancer, our study highlights the importance of considering cancer risk on a continuum based on the allele (GPV) rather than binary based on the gene. Currently, cancer penetrance estimates are anchored to the “typical” cancer risks attributed to a specific gene. Yet, given the variations in risk based on specific GPVs, such as the established differences in breast cancer risks for the *CHEK2* 1100delC GPV compared to the *CHEK2* I157T GPV genotypes, it is important to account for these differences when estimating risks. Beyond *CHEK2* GPVs, multiple factors are used to estimate breast cancer risk using programs such as CanRisk, including family history, hormonal and lifestyle factors, breast density, and polygenic risk scores [21]. Yet genotype-based risks for GPVs that deviate from the “typical” cancer risks attributed to a specific gene, such as the *CHEK2* I157T GPV, cannot be used in these risk models, which poses challenges to translation and incorporation of this information in clinic to generate risk estimates for patients. Consequently, there remains an unmet need to develop tools to estimate inherited cancer gene risks as a continuous rather than categorical variable. There remains a need to refine cancer risks by incorporating polygenic risk scores among those with inherited cancer GPVs, which may serve to either increase or decrease existing risks. Ultimately, refining risks could further refine cancer risk management. Interestingly, the *CHEK2* I157T variant is not reported on standard germline inherited cancer panels in most countries outside of the United States, specifically because it does not reach the risk threshold to independently guide care [22]. Ultimately, anchoring guidance for cancer risk management to the level of individual risk rather than to a gene may enhance risk-informed care.

The truncating 1100delC and missense I157T *CHEK2* GPVs are the most common GPVs identified in the *CHEK2* gene among European ancestry populations, with population frequencies of 1.5% and 5%, respectively [5,23,24,25]. Consequently, the high carrier frequency has enabled GPV-specific breast cancer risks to be estimated for these specific variants, recognizing that this approach is not possible for most *CHEK2* GPVs due to the limited sample size of individuals with specific GPVs. Another *CHEK2* missense GPV, c.1283C>T (p.Ser428Phe), herein referred to as “S428F”, is established to also confer lower breast cancer risks, with a previously reported odds ratio of 1.26 (95% CI 0.76–2.12; *p* = 0.37) [14], consistent with estimates from other studies [10,12]. Yet the lower risks of breast cancer observed for the missense *CHEK2* I157T and S438F variants cannot be presumed for other missense variants. In fact, in a study of over 6000 *CHEK2* carriers (excluding the *CHEK2* I157T and S428F GPVs, which were analyzed separately), there were no differences in breast cancer risk between missense and truncating *CHEK2* GPVs [3]. Moreover, estimates of breast cancer risk specifically for *CHEK2* I157T and S428F GPVs spanned from none to minimal for these variants, based on risks for invasive versus in situ breast cancers. In contrast, the common missense variant c.349A>G (p.Arg117Gly), herein referred to as “R117G”, was reported to be associated with breast cancer risks similar to truncating variants, with an odds ratio for breast cancer exceeding 2 in multiple studies [7,26], demonstrating that some missense variants are associated with clinically relevant breast cancer risk. For the less common missense variants, it remains a challenge to determine which variants are associated with clinically relevant cancer risk and which are not. It is possible that functional assays may guide clarification of risks in the future, as suggested by a recently published quantitative functional assay for *CHEK2* missense variants, which suggested that variants demonstrating a similar functional impact to the R117G variant may confer the same level of cancer risk [27].

The current study has several strengths, including recruitment across diverse settings and providers, with a relatively large sample size of females with *CHEK2* GPVs, alongside a rigorous collection of genetic test results and self-reported cancer risk management and treatment data verified where possible through medical records. Despite these strengths, there remain some limitations of our work, including limited participant diversity, a lack of information to definitively determine whether surgeries to remove breasts and/or ovaries were based on *CHEK2* status versus part of the medical treatment for breast cancer, and a lack of data to determine if compliance with annual breast MRIs was met.

Overall, findings from our study suggest that females with the *CHEK2* I157T GPV may be receiving care beyond that needed based on breast cancer risks and that recommended by current guidelines. Both females with *CHEK2* 1100delC and I157T *CHEK2* GPVs may be receiving risk-reducing surgeries to prevent both breast and ovarian cancer, which may again be care beyond that currently recommended. Consequently, our results suggest that some treating healthcare providers may manage these two *CHEK2* variants similarly, identifying an opportunity for education to improve adherence to risk-informed care.

## Figures and Tables

**Table 1:** Participant Demographic Information.

	Overall *N* = 150	
	*n*	%
Non-Hispanic White	143	95.3%
Married or Cohabitating	117	78.0%
College Graduate	92	61.3%
Private Insurance	110	73.3%
Employed Full Time	89	59.3%
Annual Income > USD 50,000	100	66.7%

**Table 2:** Differences in Breast Cancer History and Cancer Risk Management Practices Across Females with Truncating *CHEK2* c.1100delC (p.Thr367fs) and Missense *CHEK2* c.470T>C (p.Ile157T) Variants.

	*CHEK2* c.1100delC (p.Thr367fs) *N* = 104		*CHEK2* c.470T>C (p.Ile157T) *N* = 46		*p*-Value
	*n*	%	*n*	%	
Mean Age of Breast Cancer Diagnosis	49		55		0.093
Mean Age at Genetic Testing	51		49		0.557
Personal History of Breast Cancer	46	44.2%	15	32.6%	0.181
Family History of Breast Cancer	70	67.3%	31	67.4%	0.992
Personal History of Other Cancer	27	26.0%	7	15.2%	0.147
HR+ (i.e., ER+ and/or PR+) Receptor Status ^1^	38	82.6%	12	80.0%	0.819
Stage ^1^					-
Stage 0	8	17.4%	1	6.7%	
Stage 1	12	26.1%	5	33.3%	
Stage 2	14	30.4%	6	40.0%	
Stage 3	1	2.2%	2	13.3%	
Stage 4	3	6.5%	-	-	
Unknown	8	17.4%	1	6.7%	
Contralateral Prophylactic Mastectomy ^1^	23	50.0%	6	38.9%	0.501
Bilateral Prophylactic Mastectomy ^2^	8	13.8%	2	6.5%	0.296
Breast MRI with Breast Cancer ^3^	15	65.2%	5	55.6%	0.612
Breast MRI without Breast Cancer ^4^	22	44.0%	13	44.8%	0.943
Bilateral Salpingo-oophorectomy ^5^	11	24.4%	4	22.2%	0.852
Prophylactic Bilateral Salpingo-oophorectomy ^6^	10	17.5%	5	16.7%	0.918

^1^ Out of those with a history of unilateral breast cancer (1100delC *N* = 46; I157T *N* = 15). ^2^ Out of those without a history of breast cancer (1100delC *N* = 58; I157T *N* = 31). ^3^ Out of those with a history of unilateral breast cancer who have not had a bilateral mastectomy (1100delC *N* = 23; I157T *N* = 9). ^4^ Out of those without a history of breast cancer who have not had a bilateral mastectomy (1100delC *N* = 50; I157T *N* = 29). ^5^ Out of those with a history of breast cancer, but not ovarian cancer (1100delC *N* = 45; I157T *N* = 18). ^6^ Out of those without a history of breast or ovarian cancer (1100delC *N* = 57; I157T *N* = 30).

**Table 3:** Logistic Regression Model Predicting Breast Cancer.

Predictor Variables		Estimate	SE	tStat	P
Breast Cancer	GPV (1100delC or I157T) ^1^	0.51	0.37	1.35	0.18
	Family History of Breast Cancer ^2^	−0.29	0.37	−0.79	0.43
Breast MRI	GPV (1100delC or I157T) ^1^	0.03	0.37	0.09	0.93
	Personal History of Breast Cancer ^2^	0.86	0.35	2.46	0.01
	Family History of Breast Cancer ^2^	0.33	0.37	0.89	0.38
Bilateral Mastectomy	GPV (1100delC or I157T) ^1^	1.93	0.48	1.20	0.23
	Personal History of Breast Cancer ^2^	0.58	0.43	4.54	5.71 × 10^−6^
	Family History of Breast Cancer ^2^	0.03	0.45	0.07	0.94
Bilateral Salpingo-oophorectomy	GPV (1100delC or I157T) ^1^	0.03	0.45	0.06	0.95
	Personal History of Breast Cancer ^2^	0.66	0.41	1.62	0.11
	Family History of Breast Cancer ^2^	−0.18	0.43	−0.41	0.68

^1^ The data presented are that of the GPV (Germline Pathogenic/Likely Pathogenic Variant) 1100delC. ^2^ The data presented are that of “Yes”.

**Table 4:** Logistic Regression Model Predicting Prophylactic Bilateral Mastectomy and Prophylactic Bilateral Salpingo-Oophorectomy.

Predictor		Estimate	SE	tStat	P
Bilateral Prophylactic Mastectomy	GPV (1100delC or I157T) ^1^	0.82	0.83	0.99	0.32
	Family History of Breast Cancer ^2^	0.54	0.84	0.65	0.52
Bilateral Prophylactic Salpingo-Oophorectomy	GPV (1100delC or I157T) ^1^	−0.02	0.61	−0.03	0.97
	Family History of Breast Cancer ^2^	−0.68	0.59	−1.14	0.26

^1^ The data presented are that of the GPV (Germline Pathogenic/Likely Pathogenic Variant) 1100delC. ^2^ The data presented are that of “Yes”.

## Data Availability

The original contributions presented in the study are included in the article, further inquiries can be directed to the corresponding author.
